# Light and sporadic physical activity overlooked by current guidelines makes older women more active than older men

**DOI:** 10.1186/s12966-017-0519-6

**Published:** 2017-05-02

**Authors:** Shiho Amagasa, Noritoshi Fukushima, Hiroyuki Kikuchi, Tomoko Takamiya, Koichiro Oka, Shigeru Inoue

**Affiliations:** 1 Department of Preventive Medicine and Public Health, Tokyo Medical University, 6-1-1 Shinjuku, Shinjuku-ku, Tokyo, 160-8402 Japan; 2 Faculty of Sport Sciences, Waseda University, 2-579-15 Mikajima, Tokorozawa, Saitama, 359-1192 Japan

**Keywords:** Physical activity, Sedentary, Recommendation, Guideline, Accelerometry, Elderly, Epidemiology

## Abstract

**Background:**

Men are generally believed to be more physically active than women when evaluated using current physical activity (PA) guidelines, which count only moderate-to-vigorous physical activity (MVPA) in bouts lasting at least 10 min. However, it remains unclear men are truly more physically active provided that all-intensity PA are evaluated. This population based cross-sectional study aimed to examine gender differences in patterns of objectively-assessed PA in older adults.

**Methods:**

One thousand two hundred ten community-dwelling Japanese older adults who were originally randomly selected from residential registry of three municipalities were asked to respond a questionnaire and wear an accelerometer (HJA-350IT, Omron Healthcare). The prevalence of achieving current PA guidelines, ≥150 min/week MVPA in bouts lasting at least 10 min, was calculated. Gender differences in volume of each-intensity activity (METs-hour) were assessed by analysis of covariance after adjustment for age and wear time.

**Results:**

Data from 450 (255 men, mean 74 years) participants who had valid accelerometer data were analyzed. Women were less likely to meet the guidelines (men: 31.0, women: 21.5%; *p* < 0.05). However, women accumulated more light-intensity PA (LPA) and short-bout (1–9 min) MVPA, and thus established higher total volume of PA (men: 22.0 METs-hour/day, women: 23.9 METs-hour/day) (*p* < 0.05).

**Conclusions:**

Older women were less active when evaluated against current PA guidelines, but more active by total PA. Considering accumulated evidence on health benefits of LPA and short-bout MVPA, our findings highlight the potential for the limitation of assessing PA using current PA guidelines.

## Background

It has been estimated that men are more physically active than women in almost every country for most of the age groups when evaluated based on current physical activity (PA) guidelines [1–3]. Guidelines recommend that older adults engage in at least 150 min of moderate-to-vigorous physical activity (MVPA) per week in bouts lasting at least 10 min [4], and provide no recommendations how the other time should be spent. Considering the accumulated evidence on health benefits of lower intensity [5–7] and fractionalized MVPA [6, 8], apart from long-bout (≥10 min) MVPA, it may be necessary to consider other activities when determining PA levels, such as sedentary behavior (SB), light-intensity physical activity (LPA) and short-bout (<10 min) MVPA. Higher LPA level is beneficially associated with mortality risk [7, 9–11] and sitting for a prolonged time negatively effects metabolic and cardiovascular health independent of MVPA [12–14].

The ubiquitous presence of lower intensity and sporadic PA could make them partly difficult to recall in questionnaire surveys [15], though such behaviors may be of particular importance, especially for older adults for whom it is hard to accumulate long-bout MVPA [5, 6, 16]. Even though self-report based assessment of PA, which has some limitations [17], has been widely used in a research setting [1], using a motion sensor worn on the body, or accelerometer, to objectively measure PA can now give a better and more accurate understanding of personal daily activity patterns [18].

To the best of our knowledge, it remains unclear men are truly more physically active provided that all-intensity PA are evaluated. Therefore, the aim of this study was to examine gender differences in patterns of objectively-assessed PA in older adults. Our hypothesis is that women are less active according to the current PA guidelines, but more active when assessed based on total volume of PA owing to light and sporadic PA derived from life-style related PA (e.g. housework).

## Methods

### Study design

This cross-sectional epidemiological study was conducted from February through June in 2015. The study sample was 1,210 Japanese older adults aged 70–79 years who took part in a community-based survey carried out in 2010 [19]. Age, gender, and residential area had been already obtained from the resident registries of municipalities in 2010. Participants were originally randomly selected from three areas in Japan: Bunkyo city and Fuchu city in Tokyo, and Oyama City in Shizuoka prefecture. The details of the sampling process for the 2010 survey and the locations, areas, population sizes, and population densities of each area are described in the previous study [19].

### Questionnaire data

Educational attainment (years of education), working status (working with income/ not working), living arrangement (with others/alone), driving status (driving/not driving), and self-rated health were evaluated by self-report questionnaire conducted in 2015. Body mass index (BMI) (kg/m^2^) was calculated from self-reported height and weight. Self-rated health was assessed with one item from SF-8 (Japanese version) that asked participants to rate their health. Participants chose the answer that most accurately describes from a 6-point scale: excellent, very good, good, fair, poor and very poor, to the question; “Overall, how would you rate your health during the past 4 weeks?” [20].

### Accelerometry

SB and PA were assessed using a tri-axial accelerometer, the Active style Pro HJA-350IT (Omron Healthcare, Japan), with a 60-s epoch length. Metabolic equivalents (METs) determined by the Active style Pro have been shown to be strongly correlated with METs measured by indirect calorimetry [21], and accurately estimate energy expenditure in free-living conditions [22]. In addition, validity of the accelerometry used in this study has been confirmed in older people [23]. Active style Pro HJA-350IT does not assess posture and SB was determined by acceleration. METs-based cutoff was used to define each intensity of activity: ≤1.5 METs for SB, 1.6–2.9 METs for LPA, 3.0–5.9 METs for moderate-intensity PA (MPA), ≥6 METs for vigorous-intensity PA (VPA), and ≥3 METs for MVPA [12, 24, 25].

Participants were asked to wear the accelerometer on their waist for seven consecutive days while awake, only taking it off for water-based activities (e.g., swimming and shower). We adapted the records as follows: records taken when wearing the accelerometer for at least 10 h per day were considered validated records [26], and defined as “non-wear” if no acceleration signal was observed for more than 60 consecutive minutes. Participants with data from at least four days regardless of week/weekend were included in the analysis in this study. If participants had accelerometer data with more than seven days, that was also included accordingly.

### Statistical analysis

Chi-squared test was conducted to compare participant characteristics between those who agreed to wear an accelerometer and those who did not. Gender differences between categorical variables were calculated using the chi-square test. BI-LINK PROFESSIONAL EDITION Ver1.0 (Omron Healthcare, Japan) was used to analyze accelerometer data. Descriptive analyses of the duration (min/day) and volume (metabolic equivalents x hours) (METs-hour) in SB, LPA, and MVPA were conducted, stratified by gender. Total MVPA was further divided into two types according to the length of MVPA-bout: short-bout MVPA (1–9 min) and long-bout MVPA (≥10 min). Long-bout MVPA was defined as 10 or more consecutive minutes above moderate intensity threshold, on the condition that one or two minutes of interruption was allowed [8, 26]. The total minutes per week of long-bout MVPA was also calculated and prevalence of achieving more than 150 min/week (i.e., meeting the current PA guidelines) was obtained.

LPA time was normally distributed. As daily minutes of SB and MVPA were not normally distributed (Shapiro-Wilk test: *p* < 0.05), median and quartiles (25%, 75%) as well as mean and standard deviation (SD) were calculated. To eliminate the effect of different wear time between genders, we also described the percentage of wear time in each activity [i.e., PA time (min/day) /total wear time (min/day)*100]. Gender differences in each activity time were examined using t-test or Mann-Whitney U test, as appropriate. Total volume of each activity (METs-hour) per day was assessed by analysis of covariance (ANCOVA) after adjustment for age and wear time. Models were re-run using additional adjustment for sociodemographic factors (residential area, living arrangement, working status, educational attainment, and driving status). The significance level was set at *p* < 0.05. All statistical analyses were conducted using IBM SPSS Statistics version 21 (SPSS Inc., Tokyo, Japan).

## Results

### Participant enrolment and characteristics

Of the 1,210 surveyed, 988 older adults completed the questionnaire (response rate: 81.7%), and 478 of those agreed to wearing the accelerometer. However, 28 were excluded for; not meeting wearing time criteria (i.e., wearing at least four days of ≥10 h/day) (*n* = 7), refusal to wear or unreturned accelerometer (*n* = 15), and system error (*n* = 6). Thus, a total number of 450 older adults (men: 255, women: 195) was included in this study. When comparisons of participant characteristics were made between those who agreed to wear an accelerometer and those who did not, significant differences were found in gender (the percentage of agreement; men: 55.6%, women: 47.7%) and educational attainment (<13 years: 48.6%, ≥13 years: 57.3%). In analyses stratified by gender, significant differences between those who agreed to wear an accelerometer and those who did not were observed in educational attainment (<13 years: 50.2%, ≥13 years: 62.2%) and self-rated health (excellent: 65.0%, very good: 63.0%, good: 56.1%, fair: 54.7%, poor: 38.9%, and very poor: 0.0%) in men while no differences between them were observed in women.

The mean age was 74.3 ± 2.9 years in men and 74.4 ± 2.8 years in women and proportion of participants did not significantly vary by residential area (Table 1). Most of the study population was living with others, <25.0 BMI. There were statistically significant gender differences in educational attainment, working status, self-rated health, driving status (*p* < 0.05).

**Table 1:** Participant characteristics

	Men (*n* = 255)	Women (*n* = 195)	
	*n* (%)	*n* (%)	*P*-value
Age			0.924
70–74 years	132 (51.8%)	102 (52.3%)	
75–79 years	123 (48.2%)	93 (47.7%)	
Residential area			0.084
Bunkyo	78 (30.6%)	64 (32.8%)	
Fuchu	91 (35.7%)	51 (26.2%)	
Oyama	86 (33.7%)	80 (41.0%)	
Educational attainment			**<0.001**
< 13 years (Up to high school)	118 (46.3%)	132 (68.0%)	
≥ 13 years (College degree or more)	137 (53.7%)	62 (32.0%)	
Living arrangement			0.076
with others	225 (88.6%)	161 (82.6%)	
alone	29 (11.4%)	34 (17.4%)	
Working with income			**<0.001**
working	94 (36.9%)	37 (19.2%)	
not working	161 (63.1%)	156 (80.8%)	
Body mass index (BMI)			0.084
< 25.0 kg/m^2^	199 (78.0%)	164 (85.0%)	
≥ 25.0 kg/m^2^	56 (22.0%)	29 (15.0%)	
Self-rated health			**0.031**
excellent	12 (4.7%)	5 (2.6%)	
very good	58 (22.9%)	41 (21.1%)	
good	147 (58.1%)	100 (51.5%)	
fair	32 (12.6%)	36 (18.6%)	
poor	4 (1.6%)	9 (4.6%)	
very poor	0 (0.0%)	3 (1.5%)	
Driving status			**<0.001**
driving	160 (62.7%)	49 (25.3%)	
not driving	95 (37.3%)	145 (74.7%)	
Moderate to vigorous physical activity			**0.032**
< 150 min/week (not meeting guideline)	176 (69.0%)	153 (78.5%)	
≥ 150 min/week (meeting guideline)	79 (31.0%)	42 (21.5%)	

*P*-value was calculated by chi-square test. Bold indicates statistical significance (*p* < 0.05)

### Patterns of physical activity

**Fig. 1:**
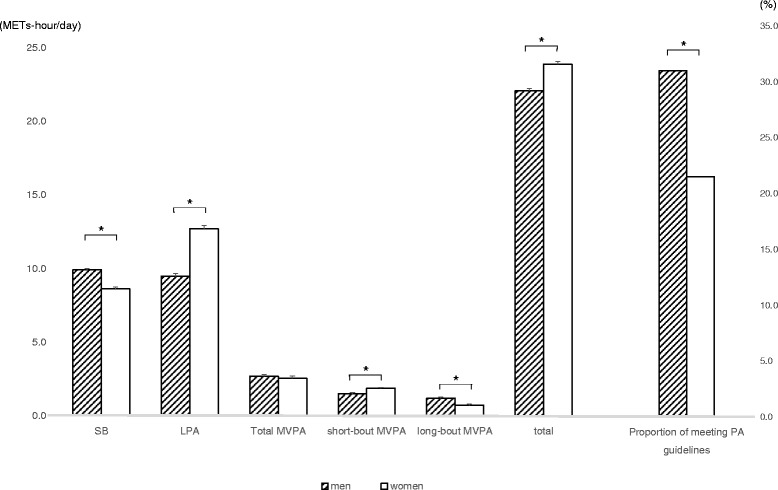
Gender differences in objectively-measured METs-hour and proportion of meeting physical activity guidelines. Abbreviations; SB: sedentary behavior, LPA: light-intensity physical activity, MVPA: moderate-to-vigorous physical activity. Estimated mean METs-hour of each activity was adjusted for age and wear time. Bars represent standard error of the mean

Mean wear time was 854.9 min/day in men and 898.6 min/day in women. Older adults spent 94.9% of wear time on SB and LPA, whereas they engaged only 5.1% for MVPA (Table 2). MPA accounted for more than 99% of MVPA in both genders. More LPA (men: 263.1 min/day, women: 365.3 min/day) and less SB (men: 548.3 min/day, women: 487.0 min/day) time was observed among women, while total MVPA time (men: 43.6 min/day, women: 46.3 min/day) did not differ by gender. Adjusting for wear time did not alter the results. When reviewing the breakdown of MVPA, more short-bout MVPA (men: 25.7 min/day, women: 33.6 min/day) was observed among women. In contrast, men engaged in more long-bout MVPA (men: 17.9 min/day, women: 12.7 min/day), thus the proportion of older adults who met PA guidelines (i.e. ≥150 min/week) was statistically higher in men (men: 31.0%, women: 21.5%) (*p* < 0.05) (Fig. 1). On the other hand, total volume of PA among women was greater compared with men [men: 22.0 (95% confidential interval: 21.7, 22.3) METs-hour, women: 23.9 (23.5, 24.3) METs-hour] (*p* < 0.05) owing to considerably higher LPA [men: 9.4 (9.0, 9.8) METs-hour, women: 12.7 (12.3, 13.2) METs-hour], and higher short-bout MVPA [men: 1.5 (1.4, 1.6) METs-hour, women: 1.9 (1.7, 2.0) METs-hour] (Fig. 1). When ANCOVA was conducted with additional adjustment for sociodemographic factors, results were unchanged. Women significantly accumulated more LPA [men: 9.3 (1.3, 1.6) METs-hour, women: 12.9 (12.5, 13.4) METs-hour] and short bout MVPA [men: 1.5 (21.7, 22.3) METs-hour, women: 1.9 (1.7, 2.1) METs-hour], while men accumulated more SB [men: 10.0 (9.8, 10.2) METs-hour, women: 8.5 (8.3, 8.8) METs-hour]and long bout MVPA [men: 1.2 (1.0, 1.4) METs-hour, women: 0.7 (0.5, 0.9) METs-hour]. Accordingly, women established higher total volume of PA than men [men: 9.4 (9.0, 9.8) METs-hour, women: 12.7 (12.3, 13.2) METs-hour].

**Table 2:** Time spent in objectively- measured physical activity by intensity in Japanese older men and women

	Men (*n* = 255)				Women (*n* = 195)				
	mean ± SD	median	(25%, 75%)	% wear time	mean ± SD	median	(25%, 75%)	% wear time	*P*-value
SB	548.3 ± 118.7	551.1	(481.2, 619.6)	64.0	487.0 ± 111.6	481.9	(418.1, 543.6)	54.2	**<0.001** ^**a**^
LPA	263.1 ± 87.4	260.6	(198.9, 319.8)	30.9	365.3 ± 90.2	359.3	(304.2, 421.0)	40.7	**<0.001** ^**b**^
MVPA									
Total MVPA	43.6 ± 30.1	39.3	(20.4, 60.6)	5.1	46.3 ± 32.8	38.8	(21.3, 65.6)	5.1	0.526^a^
Short-bout MVPA	25.7 ± 17.0	22.4	(13.0, 34.9)	3.0	33.6 ± 23.2	28.3	(16.5, 43.7)	3.7	**<0.001** ^**a**^
Long-bout MVPA	17.9 ± 21.2	9.6	(2.5, 25.9)	2.1	12.7 ± 15.9	6.3	(1.1, 19.6)	1.4	**0.012** ^**a**^

*P*-value was calculated by Mann-Whitney U test^a^ or t test^b^. Bold indicates statistical significance (*p* < 0.05).

Abbreviations: *SB* sedentary behavior, *LPA* light-intensity physical activity, *MVPA* moderate-to-vigorous physical activity

Short-bout: lasting 1–9 min, long-bout: lasting ≥10 min

## Discussion

Our findings provide a detailed analysis of PA patterns in Japanese older men and women. In summary, older women were less physically active when evaluated against current PA guidelines, but more active by total volume of PA. Considerably less time spent in SB, longer LPA and short-bout MVPA time in women were observed, which can likely be explained by lifestyle differences; Japanese women have traditionally been more responsible for most of the housework than men.

Several cohort studies have confirmed associations between longer sedentary time and higher all-cause mortality in older adults [27–29]. Epidemiological evidence is accumulating that indicates greater time spent in objectively-assessed LPA is beneficially associated with all-cause mortality [7, 9–11], cardiometabolic health [5, 30–33], mental health [31, 34, 35], and well-being [36]. As Loprinzi et al.’s study showed, for example, participants had a 16% reduced hazard of all-cause mortality [hazard ratio: 0.84; 95% CI: 0.78, 0.91] for every 60-min increase in LPA independent of objectively-measured MVPA [9]. In our sample, there was a big difference of LPA time between genders (263.1 min/day in men and 365.3 min/day in women). Approximately 100 min of LPA difference between men and women, therefore, is considered to have a meaningful impact on health outcomes. Moreover, several studies using accelerometers suggest short-bout MVPA improves metabolic health [8, 37–39]. Glazer et al.’s study [8], for example, confirmed a positive associations of short-bout MVPA with favorable cardiovascular disease risk including BMI, triglycerides, and Framingham risk score. Another study [37] indicates that accumulating MVPA in short-bout, compared with long-bout, might be just as beneficial in improvements of biologic health outcomes. In the present study, relative to men, women spent less time long-bout MVPA, but more time in short-bout MVPA (approximately 8 min). Presumably, women benefit more from short-bout MVPA than men. These activities, though not recommended by guidelines, may confer health benefits besides those well-established for MVPA [40].

With regard to total volume of PA, Manini et al. [41] found that objectively-measured (using doubly labeled water) free-living activity energy expenditure (EE) was strongly associated with reduced risk of mortality in healthy older adults, and they indicates expending EE through any activity may influence survival. Another recent study by Salonen et al. [42] reported that objectively-determined total PA volume was beneficially associated with various components of metabolic syndrome including waist circumstance, blood pressure, cholesterol, triglycerides, and fasting glucose.

For those described above, PA level and patterns specific to women may partly contribute to Japanese women’s longevity compared with men. PA level and patterns have not yet been addressed to explain why women live longer than men. One reason is that it has been estimated that men are more physically active than women in almost every country for most of the age groups when evaluated based on the current PA guidelines [1–3], where short-bout MVPA and LPA are overlooked [4].

### Implications of our findings

In this study, men who tend to achieve 150 min/week MVPA do not necessarily accumulate a greater level of LPA than women. In contrast, not meeting 150 min/week MVPA does not always correspond to a lower level of LPA as shown in women. Accordingly, looking at only MVPA become blind to the substantial benefits of PA including LPA. The results of this study suggest that evaluation criteria on PA level and public health message should be refined to include LPA in order to take PA patterns into account. In practice, we have to be careful when identifying population groups in need of interventions since there may be the discrepancy between meeting PA guidelines and total volume of PA. Older men who meet current PA guidelines but spend most of their time SB are also target of future interventions to increase LPA. For older women who are active in LPA, future research is needed to assess the health benefits by additional MVPA.

### Strengths and limitations

Our study is the novel investigation of gender differences in PA patterns including SB, LPA, and different durations of MVPA in one study which goes beyond previous studies that just examined total MVPA and long-bout MVPA [26, 43] or did not include all-intensity PA [44]. PA and SB were objectively measured by a tri-axial accelerometer, which has a validated algorithm [21, 45]. It is reported that Active style Pro HJA-350IT can be an accurate measurement for EE in free-living condition [22], although a previous study indicates that PA intensities (i.e., METs) during walking can be somewhat underestimated in older adults [23]. Using tri-axial accelerometers can provide accurate estimates [46–48], and validated the estimated EE in lower intensity PA [46, 49]. In addition, our sample is community-dwelling older adults from different area rather than “at risk” clinical groups, so findings should be generalizable to wider older adult populations. Some limitations of this study need to be considered when interpreting results. First, although accelerometers provide objective measures, they cannot accurately detect postural information (i.e., standing vs. sitting) and capture some types of PA (e.g. bicycling, water-based activities), thus these activities might be uncounted or under/overestimated [18]. However, regarding the results of questionnaire and a simple personal log by self-reported simultaneously carried out in this study, these activities accounted for only a very small proportion of total PA performed by older adults during the adopted days to the analyses. Second, we need to consider selection bias. In our sample, response rates were slightly different between men and women. There is a possibility that PA patterns may differ between those who agreed to wear an accelerometer and those who did not. Finally, the age range of our sample was relatively narrow. Our findings of this study might not be generalized to the other elderly population (e.g., ≥80 years). Further research including more wide range of older people is needed.

## Conclusions

Patterns of objectively-assessed PA were greatly differed by gender in community-dwelling older adults in Japan. Older women were less physically active than older men when evaluated against current PA guidelines, but more active by total volume of PA including LPA and short-bout MVPA. Considering accumulated evidence on health benefits of light and sporadic PA, our findings highlight the potential for the limitation of assessing PA using current guidelines.

## Abbreviations

BMI: Body mass index
EE: Energy expenditure
LPA: Light-intensity physical activity
METs: Metabolic equivalents
MVPA: Moderate-to-vigorous physical activity
PA: Physical activity
SB: Sedentary behavior
